# Teaching middle ear anatomy using a novel three-dimensional papercraft model

**DOI:** 10.1007/s00405-020-06350-8

**Published:** 2020-09-24

**Authors:** John Guy, Jameel Muzaffar, Christopher Coulson

**Affiliations:** 1 Medical School, College of Medical and Dental Sciences, University of Birmingham, Edgbaston, Birmingham, B15 2SG UK; 2 Department of Otolaryngology, Queen Elizabeth Hospital Birmingham, Edgbaston, Birmingham, B15 2TH UK

**Keywords:** Medical education, Anatomy, Middle ear, Paper model

## Abstract

**Background:**

The middle ear is a complex anatomical space which is difficult to interpret from two-dimensional imagery. Appropriate surgical knowledge of the area is required to operate, yet current anatomical teaching methods are costly and hard to access for the trainee.

**Methods:**

A papercraft 3D design involving anatomical elements added separately to a model was designed, and then peer-validated by medical students and junior doctors. Preliminary quantitative assessment was performed using an anatomical labelling questionnaire, with six students given a lecture to act as a control. Qualitative feedback was also gathered.

**Results:**

18 participants were recruited for the study. A total of 12 models were constructed by 6 medical students and 6 junior doctors. 6 medical students received a lecture only. Qualitative feedback was positive and suggested the model improved knowledge and was useful, yet timing and complexity were issues. Students scored, on average, 37% higher after completing the model, with junior doctors also improving anatomical knowledge, though these differences were not significant (*p* > 0.05).

**Conclusions:**

In this initial investigation, the model was shown to be an engaging way to learn anatomy, with the tactile and active nature of the process cited as benefits. Construction of the model improved anatomical knowledge to a greater extent than a classical lecture in this study, though this difference was not significant. Further design iterations are required to improve practical utility in the teaching environment, as well as a larger study.

**Electronic supplementary material:**

The online version of this article (10.1007/s00405-020-06350-8) contains supplementary material, which is available to authorized users.

## Introduction

The anatomy of the middle ear is intricate and conceptually complex. To operate safely, the surgeon must have adequate spatial cognition of the middle ear in three dimensions, together with knowledge of the delicate anatomy passing through or near this space. This mental model consisting of the morphology and interrelationships of anatomical structures must be conceptually adapted to accommodate different patient head angles, different approach methods, and the change in fields of view as the observer’s position is rotated around the patient’s head during surgery [1, 2]. In addition to this, related anatomy is often hidden within bony canals, meaning that the surgeon must be able to operate without seeing some structures, yet knowing where they are; identifying and drilling only in areas they know to be safe. Moreover, the observation of structures is often limited by the presence of other anatomy that can occlude the view [3].

Teaching this anatomy is challenging. Basic methods such as lectures are simple to create and distribute, but the use of passive learning limits their effectiveness [4]. More comprehensive three-dimensional methods of anatomical education exist, with dissection being the gold standard, yet there is extremely limited access especially to junior trainees and students [5]. Other models such as 3D printed temporal bones have been well described, but these can be expensive to produce in large quantities [6–10].

Limited numbers of accessible models have been described in the literature. A model by Gopalan and Menon produced an innovative model of the epitympanum from Perspex, yet this neglected the remainder of the tympanum [11]. A papercraft temporal bone was created by Hiraumi et al., which used folded compartments of paper to simulate the walls and spaces of the middle ear [12]. While serving as excellent inspiration, the model was deemed to lack sufficient detail. Other approaches included clay modelling, yet none specifically catered to middle ear anatomy [13–15].

The aims of this project were to create a model that was more accessible and practical than the traditional teaching methods, while remaining an effective educational aid.

## Materials and methods

A papercraft model was designed which included all relevant middle ear structures (see Text Document, Supplemental Digital Content 1). An instruction manual which described the structure and function of the various anatomical parts was also produced to ensure that participants could construct it independently.

**Fig. 1:**
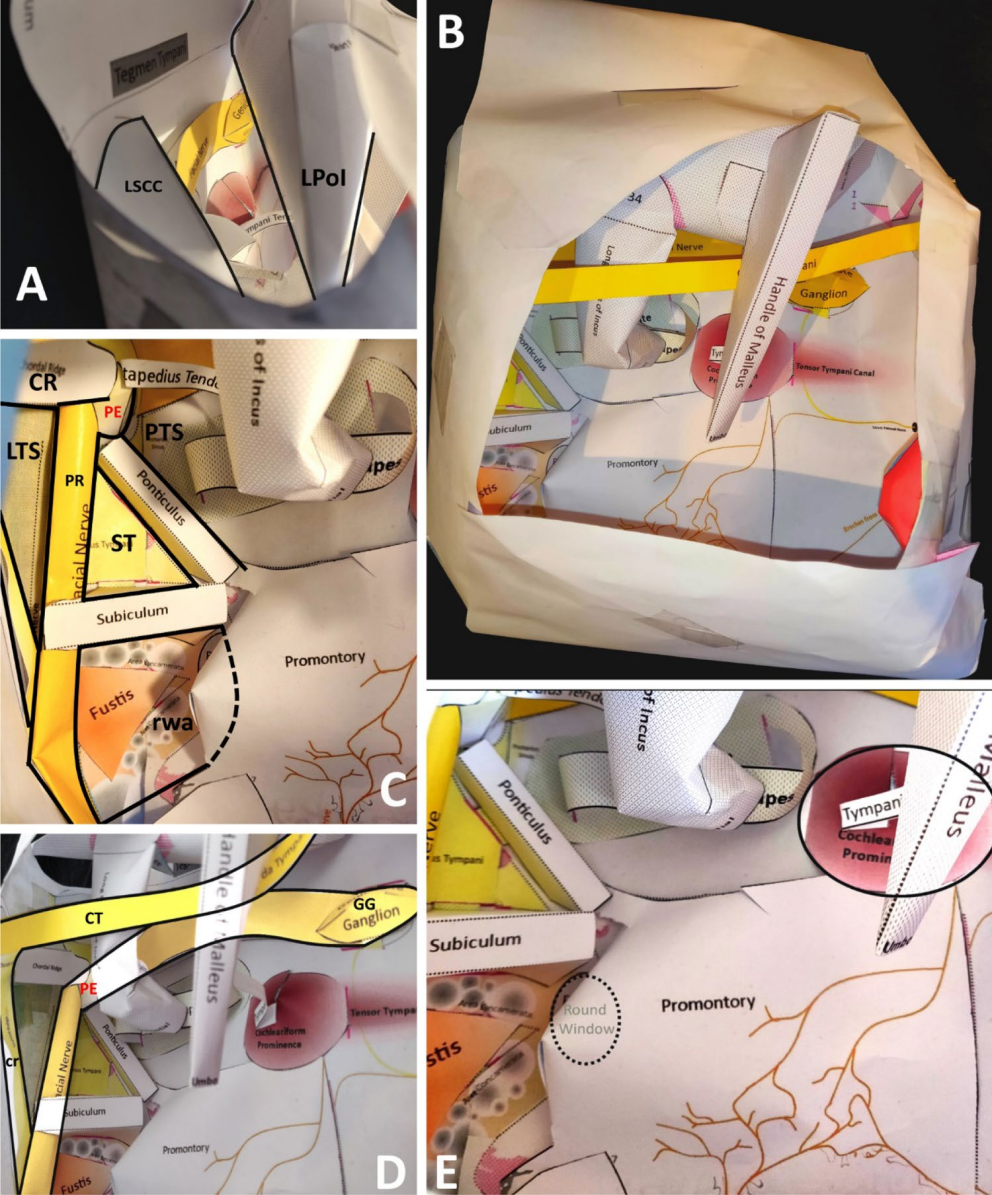
Images of the papercraft model. **a** View of the model via the aditus ad antrum (*LSCC* lateral semicircular canal, *LPoI* long process of incus). **b** View of the model representing a lateral view of a right middle ear, with ossicles in situ. **c** The retrotympanum (*CR* chordal ridge, *PE* pyramidal eminence, *PTS* posterior tympanic sinus, *LTS* lateral tympanic sinus, *PR* pyramidal ridge containing facial nerve, *ST* sinus tympani, *rwa* round window area). **d** Highlighted facial nerve (*CT* chorda tympani, *GG* geniculate ganglion, *PE* pyramidal eminence). **e** View of the mesotympanum. The cochleariform prominence and round window are highlighted

The model was designed, such that it could be printed onto normal A4 paper. The various components were then cut out from their surrounds and adhered together, according to instructions, to create a flat base. Additional detail was then added by attaching anatomically labelled paper parts to this base using tabs. This 2D ‘net’ was then folded into a box like shape. It assumed a stylistic representation of the anatomy, aiming to conserve the general location and relationship between objects of interest, as opposed to a lifelike representation. Colour was used to identify different anatomical areas, objects, and spaces. Images of the model can be seen in Fig. 1.

The educational efficacy of the model was then evaluated in a small preliminary assessment. In the first arm of the assessment, 15 medical students had their baseline knowledge measured with an anatomical labelling questionnaire (see Text Document, Supplemental Digital Content 2). The participants were then randomised into an intervention and control group. The intervention group were asked to build the teaching model, and the control group were given a lecture detailing the anatomy of the middle ear. The participants’ anatomy knowledge was then reassessed. In the second arm, 10 junior doctors were asked to construct the model, and the change in baseline knowledge was also assessed with the repeated questionnaire. Qualitative feedback was also gathered from both groups.

Ethical approval was sought from relevant authorities prior to the project. Appropriate and informed consent was sought from participants before the study took place. All participant contributions were kept strictly confidential and blinded from the project lead. All collected data and feedback forms were anonymously transcribed and then destroyed. No participant identifiable information was collected at any point during the study.

The data were analysed using SPSS software Table 1.

**Table 1:** Participant recruitment

	Medical students	Junior doctors
Participants recruited	15	10
Dropped out	3	4
Completed model construction	6	6
Received lecture	6	0

## Results

**Fig. 2:**
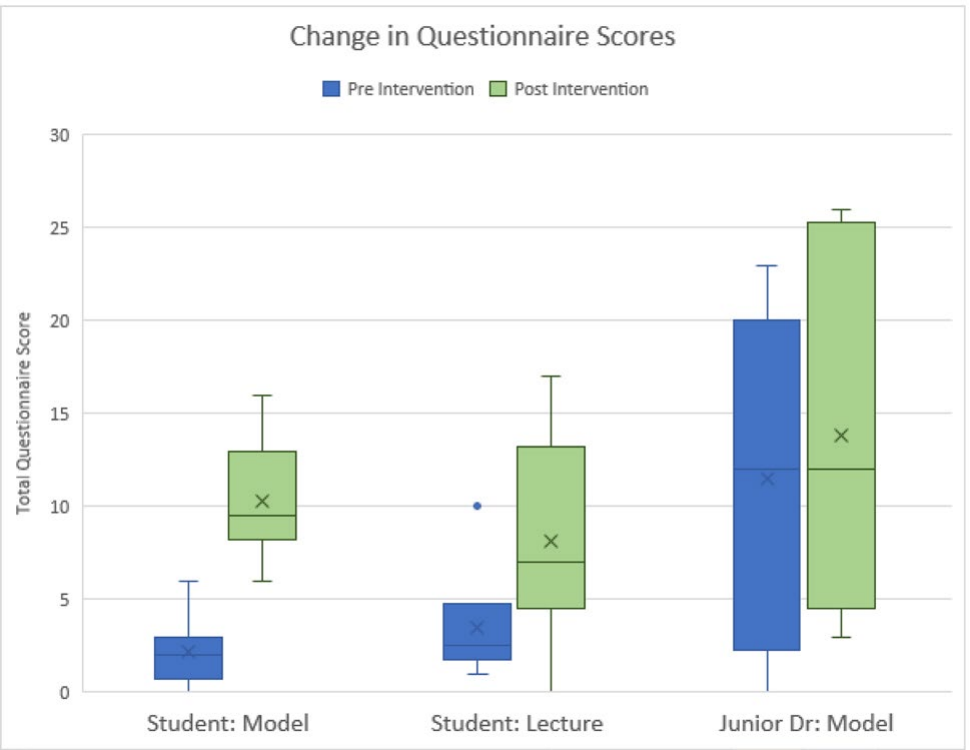
Changes in test score pre- and post-educational intervention

**Fig. 3:**
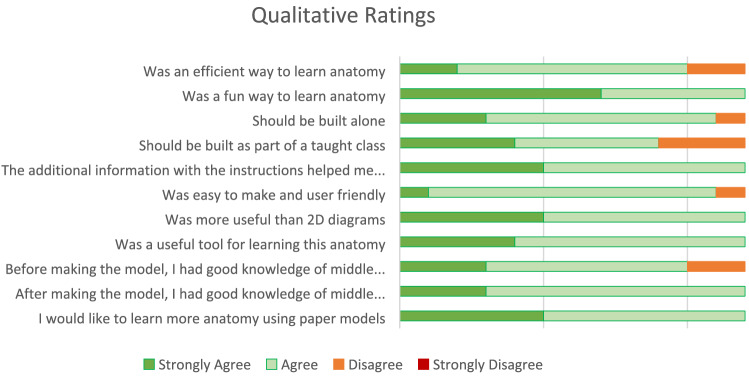
Summed responses from qualitative feedback collected from all participants who built the model

In the objective labelling test, the model group was shown to have higher post-test mean scores than the lecture group, though this difference was not significant. As seen in Table 2, there were no significant pre- to post-differences with either intervention for any group. There was a higher change in mean total score seen before and after the model session than was seen in the lecture session (Figs. 2, 3).

**Table 2:** Knowledge baseline changes following interventions

Student: lecture	Change in score			
	Q1	Q2	Q3	Total change
Mean score change	0.17	1.33	3.17	4.67
SD	0.7528	1.9664	2.5626	3.7238

Student: model	Change in score			
	Q1	Q2	Q3	Total change
Mean score change	0.83	2.08	3.50	6.42
SD	1.2247	1.3292	1.7224	2.9944
Percentage increase	+ 388%	+ 56.3%	+ 10.4%	+ 37.5%
*P* value (two tailed)	0.13978	0.24802	0.59462	0.15544
	Cohen’s d (effect size)			1.25320

Junior Dr: model	Change in score			
	Q1	Q2	Q3	Total change
Mean score change	0.33	0.17	1.83	2.33
SD	1.0328	0.9832	1.4720	2.8048

There is heavy overlap in score before and after the junior doctors constructed the model. Large standard deviations indicated a wide variance in middle ear anatomy knowledge. The mean total score was higher after the model making session, though this was not significant.

The mean number of points gained per person per question shows that participants gained more points on the diagrammatic representation of the middle ear than on the endoscopic images. This was raised in qualitative feedback, with comments suggesting that endoscopic pictures are “too complex” and “disorientating”.

## Discussion

The use of lectures and video teaching is standard practice throughout medical education, yet recent studies have shown that this passive learning is inferior to active learning, especially when teaching anatomy [4]. The gold standard in anatomical education is human dissection, allowing free exploration of structures in vivo while also simulating surgical technique. However, the difficulty in acquiring, storing, and providing these specimens to students limits their usefulness, rendering this methodology applicable only to senior surgical trainees [5, 16, 17]. 3D printed temporal bones are easier to procure, but are still not widely available, and necessitate similar surgical equipment to drill and dissect. These also suffer from the lack of adequate labelling of anatomy, rendering them uninterpretable to the newcomer without senior support. Virtual models have become more advanced in recent years, with their educational use well demonstrated [18–21]. The lack of tactile feedback can, nevertheless, reduce their utility, and while requiring less expensive equipment than real-life dissection, the prerequisite of a powerful computer can limit implementation in developing countries or in large classrooms [22].

This papercraft model was more accessible and practical than previously described models, being simple to print and requiring no specialist equipment. It could be constructed relatively quickly and in any given location, making it ideal for classroom teaching sessions. It also required little pre‐existing knowledge, as the structure and function of the anatomy was taught during construction. This makes the model ideal for students in the early stages of training, or for resource-scarce locations.

This initial study indicated that participants improved on their baseline knowledge of middle ear anatomy after they constructed the model, though these differences were not significant. In addition, a large effect size was seen, showing the model improved knowledge to a greater extent than a lecture, though again this was not significant. All groups of participants had improved knowledge after construction, with improvement shown in identifying structures on endoscopic imagery as well as labelling diagrams. Making the model a low-fidelity representation of the anatomy has been shown to improve the initial understanding of a complex shape, and here helped to facilitate knowledge gain at a range of training levels [23].

Qualitative feedback showed that this model was a novel and engaging way to learn anatomy, and participant confidence in their anatomical knowledge also improved. Note was made of the tactile nature of the model, with the ability to manipulate the model in 3D space and demonstrate different surgical approaches being of interest. Furthermore, participants reported that they would enjoy learning further anatomy through paper model construction, indicating that this methodology can be extended to teach a wider range of anatomy. Physical construction has been established as an effective method of learning anatomy, and the constructive nature of this model was well received [22].

The preliminary nature of the validation study led to inherent limitations. Though differences were seen, they were not statistically significant, which may be due to the small-sample sizes utilised. They were also allowed more time with their educational material than the lecture group, and also received more contact from the session administrator as the model was built, with more time to ask questions and raise concerns. The immediate nature of the testing also fails to examine the knowledge that is retained by the subjects, and further research must demonstrate the long-term results of this educational modality.

There were several issues with the model itself that must be addressed before further validation. Though feedback from the model was generally positive, of concern was difficulty in construction. The intricacy of constructing this model meant that construction times varied. Even after all components had been printed and cut from the supporting paper, the model took around 2 h for each participant to complete in a relaxed atmosphere. This effect was seen in the validation study, where several participants were not able to complete the model due to timing issues. The lecture, which improved knowledge by not significantly less, required around half the time. However, participants perceived the model as an efficient and enjoyable way to learn anatomy, suggesting that the model may be worth the extra time required. Further development, including varying paper thicknesses for certain structures and prior scoring and perforating the paper, will likely lead to an easier to build model.

In addition, the model had limited relation to in vivo anatomy. Some participants reported that they had learned the function and shape of structures, but not the name, indicating a more thorough integration of anatomical teaching into the construction may be needed. Diagrams of the middle ear were better labelled than endoscopic imagery, perhaps indicating a lack of relationship to in vivo anatomy, although this may be a function of the questionnaire used. Possible future research could utilise models constructed from a medium with higher fidelity, such as 3D printed plastic or clay, both of which have been employed for anatomical teaching in the previous studies. Refining the design to more closely approach anatomical reality would result in a more useful product for the surgical trainee. It would also be useful to quantitatively compare a paper model with the other models, and even against temporal bone dissection, to further explore its educational effect against the current gold standard.

The creation of this model of the middle ear showed some clear benefits over classical methods of teaching anatomy, such as improved accessibility and practicality in the teaching environment, as well as a non-significant increase in anatomical knowledge.

## Electronic supplementary material

Below is the link to the electronic supplementary material.

Supplementary file1 (DOCX 270 kb)

Supplementary file2 (DOCX 20 kb)
