# Supplementary material for: Teaching middle ear anatomy using a novel three-dimensional papercraft model
Source: Eur Arch Otorhinolaryngol. 2020 Sep 24;278(8):2769–74. doi: 10.1007/s00405-020-06350-8 (PMC8266719; doi:10.1007/s00405-020-06350-8)
Supplement: Supplementary file 1 — Supplementary file1 (DOCX 270 kb) [file 405_2020_6350_MOESM1_ESM.docx]

Middle Ear Anatomy Assessment


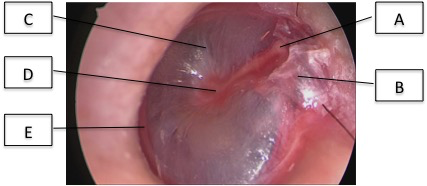


1 Please name anatomical structures as indicated in the picture above:

| A: Short process of malleus |
| --- |
| B: Shrappnell membrane (Pars flaccida) |
| C: Pars tensa |
| D: Umbo |
| E: Annulus |


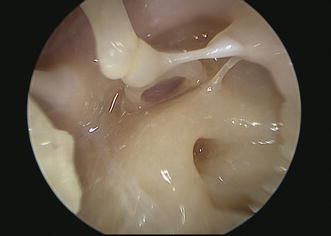


**G**

**A**

**H**

**B**

**C**

**I**

**D**

**E**

**F**

2 Please name anatomical structures as indicated in the picture above:

| A: Facial Nerve |
| --- |
| B: Pyramidal Eminence |
| C: Stapedius Tendon |
| D: Ponticulus |
| E: Long Process of Incus |
| F: Round Window Niche / Fustis |
| G: Stapes Footplate |
| H: Cochleariform Prominence / Tensor Tympani |
| I: Promontory |

**G**

**F**

**O** Promontory

**M** Promontory

**L**

**K**

**J**

**I**

**H**

**E**

**D**

**C**

**B**

**A**


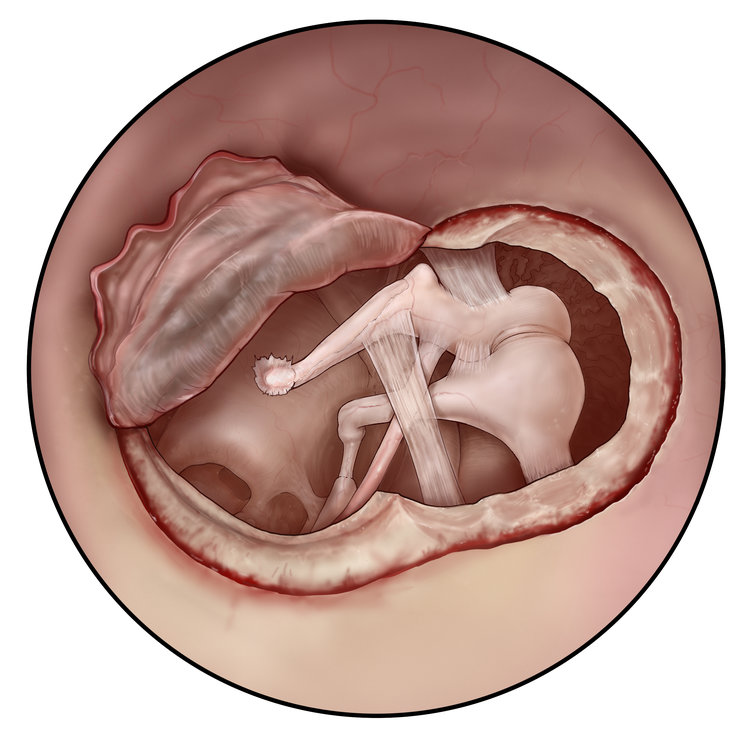


| A: Malleus Head |
| --- |
| B: Neck of Malleus |
| C: Manubrium |
| D: Umbo |
| E: Tensor Tympani Tendon |
| F: Short Process of Incus |
| G: Chorda Tympani |
| H: Long Process of Incus |
| I: Pyramidal Eminence |
| J: Ponticulus |
| K: Sinus Tympani |
| L: Subiculum |
| M: Round Window Niche |
| O: Finiculus |
