# Supplementary material for: Teaching middle ear anatomy using a novel three-dimensional papercraft model
Source: Eur Arch Otorhinolaryngol. 2020 Sep 24;278(8):2769–74. doi: 10.1007/s00405-020-06350-8 (PMC8266719; doi:10.1007/s00405-020-06350-8)
Supplement: Supplementary file 2 — Supplementary file2 (DOCX 20 kb) [file 405_2020_6350_MOESM2_ESM.docx]

List of Included Anatomical Content

Roof

**Tegmen Tympani**

Floor

**Jugular bulb**

‘Aperture’ for tympanic branch of glossopharyngeal nerve

Lateral Wall

**TM** opening

Posterior Wall

**Aditus ad antrum**

**Pyramidal Eminence**

With emergence of stapedius

**Descent of Facial Nerve**

Curve of canal behind styloid eminence

Emergence of Chorda Tympani

**Lateral Curve of Semicircular Canal**

Joining with medial wall

**Facial Recess**

Medial Wall

**Sinuses**

Sinus Tympani

Sinus Subtympanicus

Posterior Sinus tympani / Subpyramidal Space

Round Window area

Fustis

Tegmen of RW

**Windows**

Oval Window

Round Window

**Promontory**

Tympanic plexus / Jacobsen’s

**Bony Bridges**

Ponticulum

Subiculum

Funiculus

Tympanic part of bony facial nerve canal

Anterior Wall

**Eustachian Tube**

**Cochleariform Prominence**

**Tensor Tympani** muscle attachment

Curve of Internal Carotid Artery

Additional Objects

**Facial Nerve** – part of both medial and posterior walls

First genu

Geniculate ganglion

Emergence in medial wall from beneath cochleariform prominence and curve

pyramidal eminence

Descent behind styloid prominence

Emergence of Branches

**Chorda tympani**

**Muscle Tendons**

Tensor Tympani

Stapedius

**Ossicles**

Malleus

Incus

Stapes

Chorda Tympani

From posterior to anterior canaliculus

Crossing behind malleus
